# A Rare Case of Plasma Cell Cervicitis in a 39-Year-Old Female Patient: Early Clue to Looming Threats

**DOI:** 10.7759/cureus.87954

**Published:** 2025-07-14

**Authors:** Sadaf Ahmad, Dona Maria George, Nirmala Jayasankar, Annapurneswari Subramanyan

**Affiliations:** 1 Department of Histopathology, Apollo Cancer Centre, Chennai, IND; 2 Department of Histopathology and Cytology, Apollo Hospitals, Chennai, IND; 3 Department of Obstetrics and Gynaecology, Apollo First Med Hospitals, Chennai, IND; 4 Department of Pathology, Apollo Hospitals, Chennai, IND

**Keywords:** cervix, high risk hpv, hpv, plasma cells, vaccination

## Abstract

Cervical cancer is largely preventable through a combination of strategies like regular screenings, such as Papanicolaou (Pap) smears and human papillomavirus (HPV) testing, which can detect precancerous changes early, allowing for timely intervention. Additionally, the HPV vaccine significantly reduces the risk of developing cervical cancer by protecting against the high-risk strains of the virus that cause the majority of cases. Plasma cell cervicitis is characterized by plasma cell infiltration in the cervical stroma and can arise from infections, autoimmune conditions, or irritants. It is associated with high-risk HPV types 16 and 18 and features mixed inflammatory cell collections histologically. Our patient was a 39-year-old young woman who presented with on-and-off spotting per vaginum. Clinical examination and radiological examination were nonspecific. Microscopy showed polypoidal cervical tissue lined by endocervical epithelium. Stroma showed dense sheets of plasma cells along with a few lymphocytes. High-risk HPV DNA testing was negative. The patient was managed medically and vaccinated for high-risk HPV, and the follow-up Pap smear was free of plasma cells. This report highlights a rare case of plasma cell cervicitis in a young female, emphasizing the need for screening for high-risk HPV and regular cytological screening in the future so that cervical cancer can be diagnosed and prevented at the earliest.

## Introduction

Plasma cell cervicitis is a distinctive form of cervicitis characterized by the infiltration of plasma cells in the cervical stroma. This condition is part of a broader spectrum of cervical inflammation, which can arise from various causes, including infections, autoimmune processes, and irritants. Understanding plasma cell cervicitis involves exploring its etiology, pathophysiology, clinical manifestations, diagnostic methods, and management strategies. Oncogenic human papillomavirus (HPV) 16 and 18 infections have been linked to plasma cell cervicitis [1-3]. Histologically, mixed inflammatory cell collections and dense plasma cell collections are observed. Solitary plasmacytoma and plasma cell granuloma are examples of differential diagnoses. Here, we are reporting a rare case of plasma cell cervicitis in a young female patient.

## Case presentation

A 39-year-old female patient presented with on-and-off spotting per vaginum prior to periods for three months. There was no other significant medical history in the past.

An ultrasound scan of the abdomen and pelvis showed a normal upper abdomen, a normal uterus with an endometrial thickness of 6.4 mm, and a normal right ovary. Two physiological cysts were seen in the left ovary, and the cervix showed a few nabothian cysts. On colposcopic examination, a small subcentrimetric cervical polyp was found.

**Figure 1:**
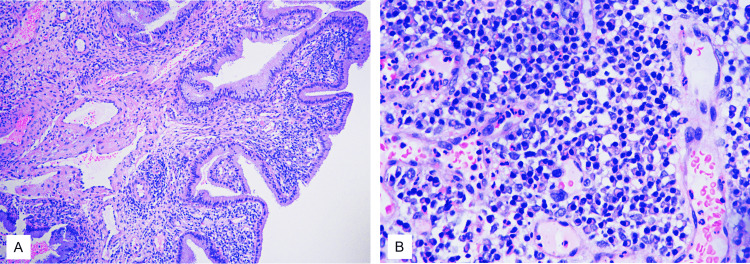
Microscopy showing polypoidal tissue lined by endocervical epithelium; stroma showing dense sheets of plasma cells along with few lymphocytes H&E: (A) 40X, (B) 400X

The patient underwent a cervical polypectomy. Histopathological examination showed tan-brown polypoidal fragments measuring 0.4-0.6 cm. Microscopy showed polypoidal tissue lined by endocervical epithelium. Stroma showed dense sheets of plasma cells along with a few lymphocytes. Focal thick-walled blood vessels were seen. Areas of ulcerations and granulation tissue were noted. The overlying epithelium showed degenerative atypia. There was no evidence of atypia or malignancy (Figure 1).

**Figure 2:**
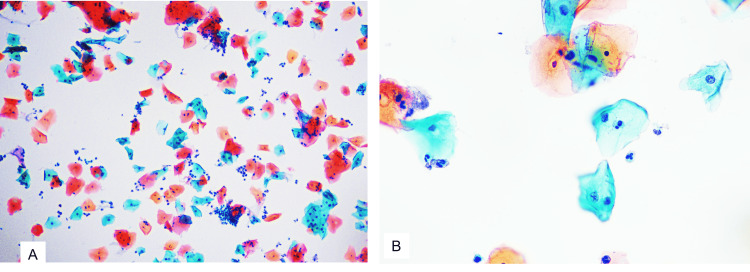
Papanicolaou (Pap) smear showing superficial and intermediate squamous cells with no intraepithelial lesion or malignancy with neutrophilic inflammation A: 100X, B: 400X

A diagnosis of plasma cell cervicitis was made. HPV DNA testing was done using real-time polymerase chain reaction (PCR) and showed negative for 14 high-risk HPV, including HPV 16 and 18. The patient was given clindamycin 300 mg orally two times a day for seven days and was symptomatically better. She was vaccinated for high-risk HPV after histopathological and molecular workup was done, approximately 15 days after presentation at the hospital. Follow-up Papanicolaou (Pap) smear test, done two weeks after vaccination, was negative for intraepithelial lesion or malignancy, with few scattered neutrophils. No plasma cells were seen in the smear (Figure 2).

## Discussion

In 2022, cervical cancer ranked as the fourth most prevalent cancer among women, with approximately 660,000 new cases and 350,000 deaths reported globally [4]. Countries with effective screening programs utilizing conventional Pap cytology have seen significant declines in mortality over the past 50 years. High-risk HPV genotypes, such as 16 and 18, are linked to nearly all cases of cervical precancer and cancer. Consequently, high-risk HPV nucleic acid testing of cervical cytology samples provides greater sensitivity and negative predictive value for detecting these conditions, particularly in women over 30, and is, therefore, recommended as an adjunct to cytology for women aged 30 and older [5].

Cervicitis is characterized by inflammation of the columnar epithelium of the uterine endocervix. Infections often cause acute cervicitis, while chronic cervicitis typically stems from non-infectious factors. Chronic plasma cell cervicitis was first described by Qizilbash in 1974 [3]. The literature review showed very few cases of plasma cell cervicitis being reported. Association of plasma cell cervicitis with oncogenic HPV 18 infection was found in all four reported cases in the literature [2,3]. Even though our patient had a negative molecular study for high-risk HPV, due to a lack of research on this rare entity, the exact point of contact with high-risk HPV is under question. Whether HPV infection occurs due to plasma cell cervicitis or plasma cell cervicitis leads to HPV infection has to be researched. No case on the association of plasma cell cervicitis and HPV infection leading to cervical cancer has been reported in the literature.

The etiology of plasma cell cervicitis is primarily associated with chronic inflammatory responses. Chronic infections, particularly those caused by sexually transmitted pathogens such as *Chlamydia trachomatis*, *Neisseria gonorrhoeae*, and HPV, are significant contributors to contact irritation due to seminal fluid, contraceptive substances, chemicals, or frequent douching [3]. These can lead to persistent antigenic stimulation of the immune system, resulting in a chronic inflammatory response characterized by the proliferation of plasma cells. Plasma cells are differentiated B lymphocytes that produce antibodies in response to specific antigens. Their infiltration into the cervical stroma signifies a sustained immune response. This can lead to changes in cervical tissue architecture, contributing to the symptoms and potential complications associated with plasma cell cervicitis.

Plasma cell cervicitis can have a wide range of presentations, even evoking an ulcerated cervical mass with a clinical suspicion of carcinoma cervix. Many patients remain asymptomatic, making it challenging to diagnose without routine screening or evaluation. A common presentation is with an abnormal vaginal discharge which can be mucoid, purulent, or blood-stained, similar to our case. Dyspareunia, intermenstrual bleeding, and pelvic pain can also occur [6]. During a gynecological examination, the cervix may appear erythematous and edematous, with visible discharge. These findings often lead to further evaluation, particularly if persistent or recurrent. Plasma cell cervicitis can coexist with other gynecological conditions, such as pelvic inflammatory disease and chronic endometritis, which can complicate the clinical picture and necessitate a more comprehensive diagnostic approach. Plasma cell cervicitis with high-risk HPV in all reported cases in the literature showed a higher incidence in elderly women [1-3], unlike the young patient in our case.

Diagnosing plasma cell cervicitis involves clinical assessment, laboratory tests, and histopathological evaluation. A thorough history and physical examination are crucial. Providers should inquire about sexual history, the presence of risk factors for sexually transmitted infections, and any associated symptoms. Radiology does not give any indication of plasma cell cervicitis. The definitive diagnosis is made through a biopsy of the cervical tissue. Histological analysis will demonstrate a significant increase in plasma cells within the cervical stroma. Immunohistochemical staining using CD 38 and CD138 can further confirm the presence of plasma cells, differentiating plasma cell cervicitis from other conditions such as lymphocytic cervicitis or neoplastic processes by restriction of clonality by kappa and lambda light chain restriction.

Plasma cell mucositis of the female genital tract involves not only the cervix but also the vagina and endometrium. Zoon’s vulvitis or plasma cell vaginitis is a rare and chronic inflammatory disorder characterized by red to orange erythematous plaques, primarily affecting the vulvar vestibule, though it can also involve the labia minora and majora [7]. Korn et al. conducted a study on endometrial biopsies of 41 women complaining of vaginal discharge and pelvic pain and found that 10 of 22 women with bacterial vaginosis in their study had plasma cell endometritis [8].

Russell body cervicitis is a similar, but rare entity in which the cytoplasm of plasma cells demonstrates Russell bodies [9]. Plasma cells with such inclusions are referred to as Mott cells. Almost all reported cases presented with cervical bleeding, and examination showed a cervical polyp. Immunoglobulins accumulate in the endoplasmic reticulum of the plasma cells under inflammatory conditions. In immunohistochemistry, they are CD138 positive and histochemically Periodic acid-Schiff (PAS) positive. Differential diagnosis of Russell body cervicitis is plasmacytoma, differentiated by demonstration of light chain restriction by immunohistochemistry, and malakoplakia, differentiated by the presence of characteristic Michaelis-Gutmann bodies, which are basophilic structures with surrounding clear halos and are PAS positive. Signet ring cells of carcinoma have to be considered as potential mimickers and are also PAS positive. In none of the reported cases of Russel body cervicitis, an association with high-risk HPV is demonstrated.

Management strategies for plasma cell cervicitis focus on addressing the underlying causes, relieving symptoms, and preventing complications. If an infectious etiology is identified, appropriate antibiotic treatment is essential. Broad-spectrum antibiotics may be initiated empirically while awaiting culture results. If sexually transmitted infections are confirmed, targeted therapy should follow. Patients experiencing pain or discomfort may benefit from non-steroidal anti-inflammatory drugs or other analgesics [10]. Education regarding the avoidance of irritants and the use of barrier methods during intercourse may also help alleviate symptoms. Regular follow-up is vital to assess treatment response and monitor for recurrence of symptoms. If patients are symptomatic despite treatment, further evaluation for underlying conditions may be necessary. Providing education on sexual health and safe practices is crucial for preventing recurrence and addressing potential risk factors for sexually transmitted infections.

While plasma cell cervicitis itself is not typically associated with severe complications, it can lead to chronic pelvic pain or dyspareunia, significantly impacting a patient's quality of life. Additionally, chronic cervicitis may increase the risk of developing cervical dysplasia or neoplasia, especially in the context of HPV infection [6-10].

## Conclusions

Plasma cell cervicitis is a multifaceted condition that highlights the interplay between chronic inflammation and cervical health. A comprehensive understanding of its etiology, clinical features, diagnostic approaches, and management strategies is crucial for healthcare providers. By addressing this condition holistically, clinicians can enhance patient outcomes, improve quality of life, and contribute to the prevention of potential complications associated with chronic cervical inflammation. Continued research into the underlying mechanisms and long-term consequences of plasma cell cervicitis will further refine our approach to this complex condition, ensuring that patients receive optimal care and support. Regular screening by Pap smear and ensuring vaccination for high-risk HPV would be advisable for patients with plasma cell cervicitis with a negative molecular assessment of high-risk HPV.
